# The mechanism of traditional medicine in alleviating ulcerative colitis: regulating intestinal barrier function

**DOI:** 10.3389/fphar.2023.1228969

**Published:** 2023-10-09

**Authors:** Qiuyun Xu, Yuan Yao, Yongchao Liu, Jie Zhang, Liming Mao

**Affiliations:** ^1^ Department of Immunology, School of Medicine, Nantong University, Nantong, Jiangsu, China; ^2^ Basic Medical Research Center, School of Medicine, Nantong University, Nantong, China

**Keywords:** ulcerative colitis, traditional medicine, intestinal barrier function, mechanism, natural compound, decoction

## Abstract

Ulcerative colitis (UC) is an idiopathic inflammatory disease mainly affects the large bowel and the rectum. The pathogenesis of this disease has not been fully elucidated, while the disruption of the intestinal barrier function triggered by various stimulating factors related to the host genetics, immunity, gut microbiota, and environment has been considered to be major mechanisms that affect the development of UC. Given the limited effective therapies, the treatment of this disease is not ideal and its incidence and prevalence are increasing. Therefore, developing new therapies with high efficiency and efficacy is important for treating UC. Many recent studies disclosed that numerous herbal decoctions and natural compounds derived from traditional herbal medicine showed promising therapeutic activities in animal models of colitis and have gained increasing attention from scientists in the study of UC. Some of these decoctions and compounds can effectively alleviate colonic inflammation and relieve clinical symptoms in animal models of colitis via regulating intestinal barrier function. While no study is available to review the underlying mechanisms of these potential therapies in regulating the integrity and function of the intestinal barrier. This review aims to summarize the effects of various herbal decoctions or bioactive compounds on the severity of colonic inflammation via various mechanisms, mainly including regulating the production of tight junction proteins, mucins, the composition of gut microbiota and microbial-associated metabolites, the infiltration of inflammatory cells and mediators, and the oxidative stress in the gut. On this basis, we discussed the related regulators and the affected signaling pathways of the mentioned traditional medicine in modulating the disruption or restoration of the intestinal barrier, such as NF-κB/MAPK, PI3K, and HIF-1α signaling pathways. In addition, the possible limitations of current studies and a prospect for future investigation and development of new UC therapies are provided based on our knowledge and current understanding. This review may improve our understanding of the current progression in studies of traditional medicine-derived therapies in protecting the intestinal barrier function and their roles in alleviating animal models of UC. It may be beneficial to the work of researchers in both basic and translational studies of UC.

## 1 Introduction

**FIGURE 1:**
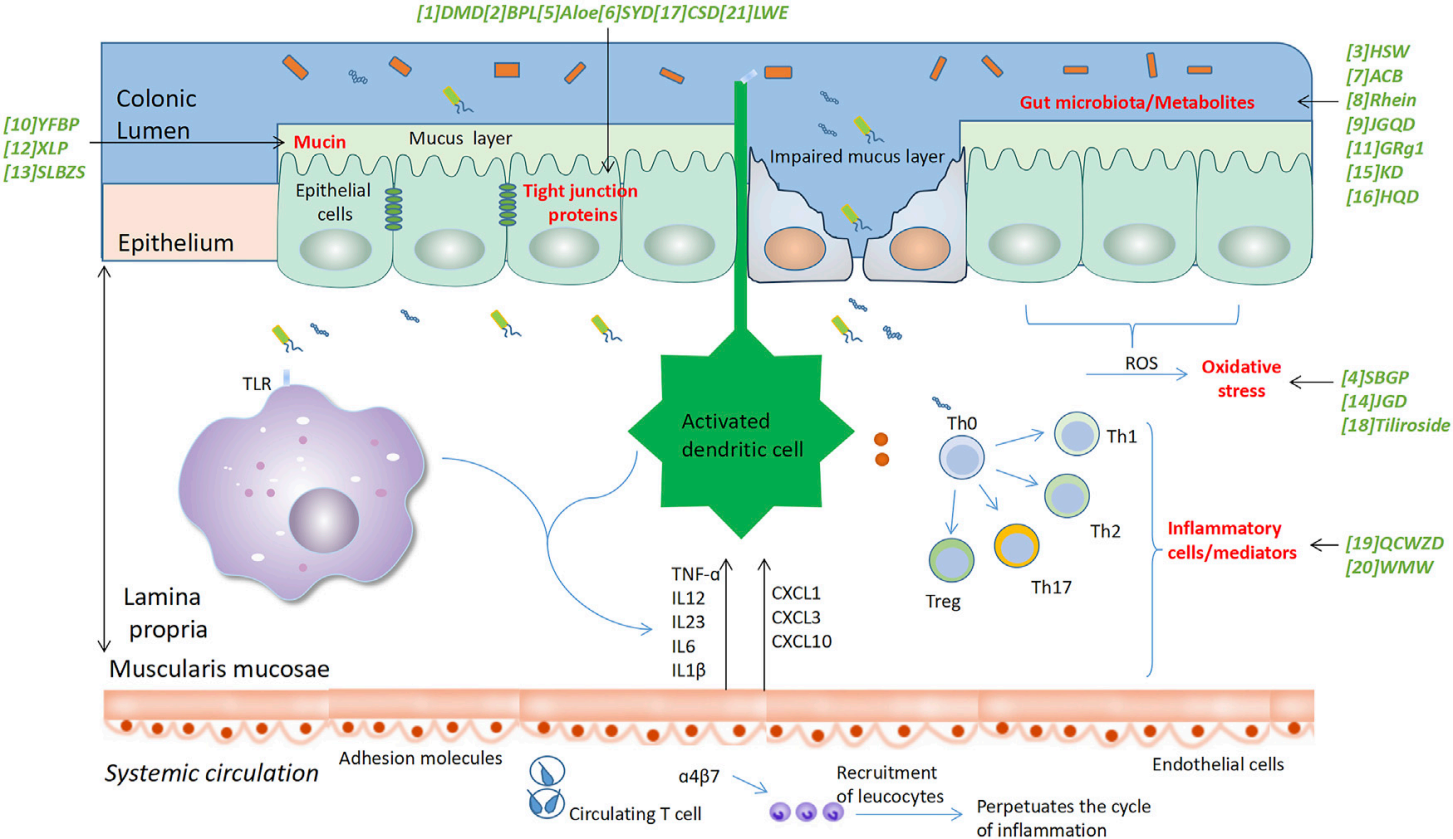
Traditional medicine-derived compounds/decoctions induce recovery of intestinal barrier function by affecting various biological processes. The initiation of UC is considered to be associated with the stimulation of particular environmental factors, such as medications, smoking, and stress, which induce impaired intestinal barrier integrity via reducing the expression of intestinal protecting proteins, such as the tight junction proteins and mucins, and triggering cell death of intestinal epithelial cells. The commensal microbes and the associated metabolites in the gut then invade the lamina propria through the damaged site of the intestinal epithelial barrier and stimulate the local inflammatory cells, such as the macrophages and dendritic cells, which then release pro-inflammatory cytokines and chemokines to recruit other types of inflammatory cells to the inflammatory sites to amplify the inflammatory responses in the gut. The invading microbes and the activation of inflammatory cells may induce oxidative stress in the gut, which causes tissue damage and further aggravate gut inflammation. Numerous compounds and decoctions derived from traditional medicine may target these biological processes and thus play a role in alleviating symptoms of UC. The decoctions/compounds were marked in green. Green arrows were used to indicate the biological processes that are affected by a decoction or a compound. Abbreviations: (1) DMD, Dahuang Mudan decoction; (2) BPL, B. pinnatum leaf; (3) HSW, Polygoni multiflori Radix (Heshouwu); (4) SBGP, Scutellaria baicalensis Georgi polysaccharide; (6) SYD, Shaoyao decoction; (7) ACB, Anemone chinensis Bunge aqueous enema; (9) JGQD, Jiawei Gegen Qinlian decoction; (10) YFBP, Yiyi Fuzi Baijiang Powder; (12) XLP, Xianglian Pill; (13) SLBZS, Shen-Ling-Bai-Zhu-San; (14) JGD, Jasonia glutinosa (L.) DC.; (15) KD, Kuijieyuan Decoction; (16) HQD, Huangqin Decoction; (17) CSD, Compound sophorae decoction; (19) QCWZD, Qingchang Wenzhong Decoction; (20) WMW, Wumei Wan; (21) LWE, Glycyrrhiza uralensis Fisch. (Licorice) water exact.

Ulcerative colitis (UC) is one of the major types of inflammatory bowel disease (IBD). It mainly affects the lower end of the digestive system, the large intestine, and the rectum (Ungaro et al., 2017; Feuerstein et al., 2019). Patients with UC display various clinical symptoms, mainly including abdominal pain, bloody diarrhea, malnutrition, fecal urgency, and tenesmus, while the progression of UC may also cause clinical manifestations in other organs (Feuerstein et al., 2019). The development of UC may promote multiple pathological changes in the intestine, such as hyperemic and granular mucosa, petechial hemorrhages, and the formation of ulcers. These alterations are accompanied by multiple histological changes, such as crypt abscess and inflammatory cell infiltration to the lamina propria (DeRoche et al., 2014). An increasing body of evidence showed that the pathogenesis of UC is associated with the interplay of multiple factors from host genetics, immunity, microbes, and the environment (Porter et al., 2020). A study by Chen et al. proposed that disruption of the circadian rhythm may also contribute to the exacerbation of colitis via regulating mitochondrial energy metabolism (Chen et al., 2022). The initiation of UC may be triggered by particular environmental factors, such as medications, smoking, and stress, which induce disruption of intestinal barrier integrity (Jensen et al., 2022). The microbes in the gut may then invade into lamina propria through the damaged intestinal epithelial barrier and stimulate the local inflammatory cells, such as the tissue-resident macrophages and intestinal dendritic cells, which subsequently release pro-inflammatory cytokines and chemokines to recruit other types of inflammatory cells, such as lymphocytes and neutrophils to the inflammatory sites. Intestinal dendritic cells and macrophages can also activate the recruited lymphocytes by antigen presentation and thereby amplify the inflammatory responses in the gut (Figure 1).

The diagnosis of UC is mainly based on the commonly presenting symptoms of UC and the continuous and diffuse colonic inflammation observed by endoscopy, while colon biopsies of patients provide further confirmation for the diagnosis (Feuerstein et al., 2019). Although recent studies have made remarkable strides in understanding the pathogenesis of UC, a specific cure or treatment for this disease has not been developed. As a common idiopathic and refractory disease, the investigation and development of therapies with high efficiency and efficacy have always been an important issue in the field of UC study. The available pharmacological therapeutic strategies are based on the application of multiple classes of drugs, mainly including 5-aminosalycilates, thiopurines, and biological agents targeting various inflammatory mediators or signaling molecules, such as tumor necrosis factor-alpha (TNF-α), integrins, and Janus kinase inhibitors. However, some patients may fail to respond to these therapies, leading to further progression of their colonic symptoms. Moreover, most treatments may cause serious adverse effects. For instance, antibody-based therapies can cause serious infection and malignancy and have been a significant concern of UC clinicians (Di Paolo and Luci, 2020; Baumgart and Le Berre, 2021). Thus, the development of new treatments with high efficacy and low adverse effects is always an important issue in studies of UC therapy.

Some recent studies suggest that numerous traditional medicine-derived decoctions or compounds show anti-inflammatory and antioxidant activities and thus manifest anti-UC effects (Andrade et al., 2020; He et al., 2021; Huang et al., 2022). These compounds or decoctions may affect the development and progression of colonic inflammation via multiple mechanisms. While one of the most important mechanisms of these therapies in colitis is to regulate the integrity of intestinal barrier function. However, no study is available to review the underlying mechanisms of the therapies in regulating intestinal barrier function. Here, we will summarize the effects of multiple herbal decoctions or compounds on colitis. Moreover, we discuss the underlying mechanisms of action, related regulators, and signaling pathways. Additionally, we provide our understanding of the possible limitations of current studies and a prospect for future investigation and development of new UC therapies.

## 2 Intestinal barrier plays a critical role in maintaining gut homeostasis

The intestinal barrier is a host protective entity composed of physical and functional barriers (Ghosh et al., 2020). The physical barrier is formed by the cooperation of the gut epithelium, the microbiota, and the mucus produced by the gut goblet cells. The intestinal epithelium consists of the layer of epithelial cells lining the gut, which forms a barrier via cell junctions, thus sealing the space between adjacent cells and blocking the entry of pathogenic microbes and other unwanted substances to exert its protective function (Peterson and Artis, 2014; Sanchez de Medina et al., 2014). Meanwhile, the goblet cell in the epithelium can produce mucin and form a layer of mucus to prevent the direct contact of potential pathogenic microorganisms to the epithelium (Yan et al., 2013; Peterson and Artis, 2014). While the commensal microbiota may protect the host by suppressing the proliferation of pathogenic microorganisms in the gut by nutritional competition and also influence epithelium function by providing energy sources, such as short-chain fatty acids, thereby assisting the proliferation of epithelial cells (Sanchez de Medina et al., 2014). The biochemical barrier consists of a variety of bioactive molecules, such as gastric acid, defensins, and Reg3γ, it also contains molecules in the regulation of microbes, such as intestinal Paneth cell produced antimicrobial peptides and gut plasma cell secreted secretory immunoglobulin A (sIgA) (Camilleri et al., 2012; Peterson and Artis, 2014; Chelakkot et al., 2018). These bioactive molecules may directly contribute to the elimination of pathogenic microbes (Schubert, 2014) or regulate the structure of microbiota (Han et al., 2022). Another ingredient of the biochemical barrier is a variety of immune cells residing in the lamina propria of the gut. These cells may produce many types of molecules, such as matrix metalloproteinase produced by activated macrophages, to exert the barrier function by regulating normal immune responses to infections (O'Sullivan et al., 2015).

The intestinal barrier plays an essential role in maintaining the gut homeostasis. Some studies have disclosed that the disruption of the barrier function or alterations in its structure is closely associated with the development of many intestinal diseases, including UC. For instance, genetic deletion of tight junction proteins can result in failure of intestinal barrier function and induce phenotypes similar to those found in UC patients (Stremmel et al., 2017). The normal function of the intestinal barrier is in tight regulation by various host and environmental factors. A nice example of the regulation of the intestinal barrier is provided by the different polarization of intestinal macrophages (Lissner et al., 2015). M1 polarization may be accompanied by the breakdown of the intestinal barrier and thereby lead to inflammatory progression of the intestine, while M2 polarization is usually associated with an elevation of tissue repair activity and remission of gut inflammation. For example, a study by Ge et al. (2023) reported that inhibition of M1 macrophage using Sotetsuflavone, a natural flavonoid derived from many herbal plants, may be beneficial to the restoration of the intestinal barrier in IBD.

Many recent studies have revealed that some traditional decoctions or herbal plant-derived compounds may have a role in protecting the maintenance of intestinal barrier integrity and thereby preventing the development and progression of experimental colitis (Andrade et al., 2020; He et al., 2021; Huang et al., 2022). The protective effects of the decoctions/compounds on intestinal barrier function are revealed to be associated with the activation of numerous signaling pathways, which regulate the alteration of metabolism processes and microbiota compositions. The metabolic and microbial changes can subsequently modulate gut immune homeostasis and determine the progression of colonic inflammation. Here, we provide an overview of the major findings of recent studies in the literature based on the mechanisms by which the decoctions/compounds exert the barrier protective role.

## 3 Traditional medicine-derived compounds ameliorate UC via various mechanisms

**FIGURE 2:**
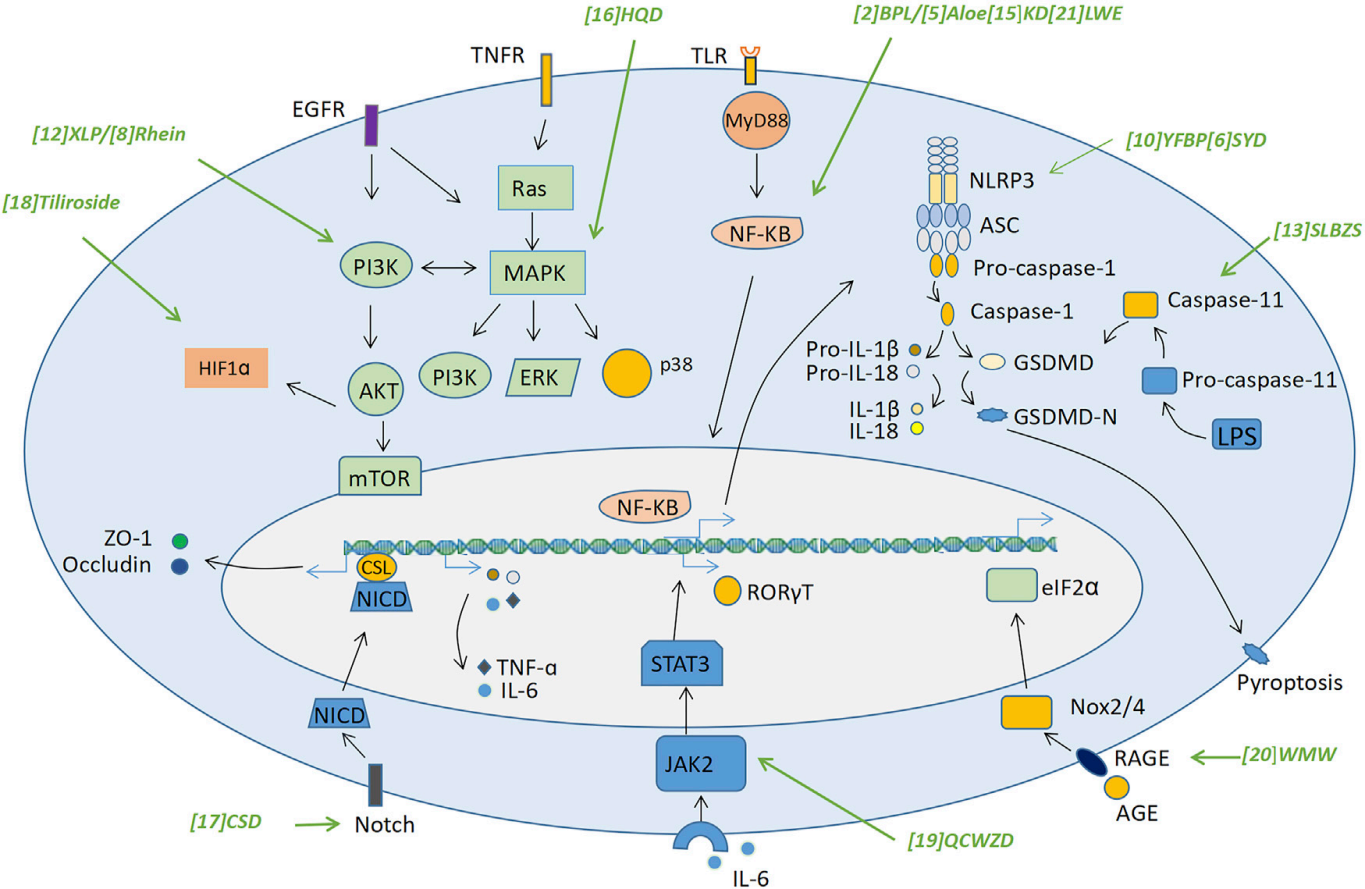
Traditional medicine derived compounds/decoctions achieve the barrier protecting effects via regulating various signaling pathways. Traditional Chinese medicine derived herbal decoctions and natural compounds alleviate UC-associated colitis through regulating a variety of signaling pathways and/or active molecules, mainly including the NF-κB/MAPK, the PI3K-AKT, the NLRP3 inflammasome/pyroptosis, the Notch, the HIF-1α, the RORγT, and the AGE-RAGE signaling pathways. These signaling pathways are thought to be associated with the damage of the intestinal barrier integrity via regulating various biological processes. The decoctions/compounds were marked in green. Green arrows were used to indicate the signaling pathways that affected by a decoction or a compound. Abbreviations: (1) DMD, Dahuang Mudan decoction; (2) BPL, B. pinnatum leaf; (3) HSW, Polygoni multiflori Radix (Heshouwu); (4) SBGP, Scutellaria baicalensis Georgi polysaccharide; (6) SYD, Shaoyao decoction; (7) ACB, Anemone chinensis Bunge aqueous enema; (9) JGQD, Jiawei Gegen Qinlian decoction; (10) YFBP, Yiyi Fuzi Baijiang Powder; (12) XLP, Xianglian Pill; (13) SLBZS, Shen-Ling-Bai-Zhu-San; (14) JGD, Jasonia glutinosa (L.) DC.; (15) KD, Kuijieyuan Decoction; (16) HQD, Huangqin Decoction; (17) CSD, Compound sophorae decoction; (19) QCWZD, Qingchang Wenzhong Decoction; (20) WMW, Wumei Wan; (21) LWE, Glycyrrhiza uralensis Fisch. (Licorice) water exact.

As mentioned above, a large body of evidence has been provided by recent studies to prove the effects of traditional decoctions, formulas, some herbal plants, and naturally occurring compounds on alleviating UC-associated colitis via various mechanisms (Andrade et al., 2020; He et al., 2021; Huang et al., 2022). In these studies, experimental animals were pre-treated with various compounds or decoctions, then colitis models were induced by the administration of various agents, such as DSS, which may cause epithelial damage and induce the entry of gut bacteria to the lamina propria. The latter then induces gut inflammation via multiple mechanisms. Here, we discussed the major mechanisms employed by traditional medicine-derived compounds/decoctions in alleviating symptoms of UC (Figure 1). Based on this analysis, we then provided an overview of the signaling pathways and/or active molecules that act as signal transduction routes by the compounds/decoctions (Figure 2). The information on the mechanisms and signaling pathways, details of the animal models of UC, and available toxicity data in the studies we mentioned were summarized in Table 1.

**TABLE 1:** Decoctions and compounds in traditional medicine protect intestinal barrier function via various mechanisms.

Compound No.	Decoction	Component	Pathway	Mechanism	Dose/concentration	Disease model	Toxicity data	Reference
**(1)**	DMD	Unidentified	Unidentified	Increases the expression of ZO-1, Occludin, and Claudin-1, the ratio of NCR + ILC3 and IL-22+ILC3; decreases the ratio of NCR-ILC3	250 mg/kg, 500 mg/kg, and 1000 mg/kg, once daily by gavage for 7 days	2% DSS-induced chronic colitis in mice	No obvious toxicity to MNK-3 (16 μg/mL) and Caco2 cells (256 μg/mL) for 24 h)	Huang et al. (Huang et al., 2022)
**(2)**	BPL	Quercetin, 3-O-α-L-arabinopyranosyl-(1→2)-α-L-rhamnopyranoside, kaempferol, 3-O-α-L-arabinopyranosyl-(1→2)-α-L-rhamnopyranoside and quercetin-3-O-rhamnopyranoside	TLRs/NF-κB	Promotes the production of mucosa protecting proteins	250 mg/kg and 500 mg/kg in DNBS-colitis; 100 mg/kg and 200 mg/kg in DSS-colitis, once daily by gavage for 7 days	DNBS-colitis in rats and DSS-colitis in mice	No obvious toxicity to mice for 14 days,(oral, 250 mg/kg and 500 mg/kg)	Andrade et al. (Andrade et al., 2020)
**(3)**	HSW	2,3,5,4′-Tetrahydroxystilbene-2-O-β-D-glucoside	Unidentified	Improves the expression of ZO-1 and occludin; balances the gut microbiota	25 mg/kg, 100 mg/kg, once daily by gavage for 7 days	3% DSS-colitis in mice	Not available	He et al. (He et al., 2021)
**(4)**	SBGP	Mannose, ribose, rhamnose, glucuronic acid, glucose, xylose, arabinose, fucose	NF-κB	Suppresses the levels of proinflammatory cytokines	50 and 200 mg/kg once a day by gastric lavage from day 1–10	3% DSS-colitis in mice	Not available	Cui et al. (Cui et al., 2021)
**(5)**	Aloe vera	Aloin A	PKC/ERK and PI3K-AKT	Upregulates the levels of mucins, suppresses the levels of IL-6, IL-1β and TNF-α, upregulates the level of IL-10	18 mg/kg and 72 mg/kg, once daily by gavage for 7 days	3% DSS-colitis in rats	No cytotoxicity to LS174T cells (80 μM for 24 h)	Shi et al. (Shi et al., 2021)
		Aloe B						
		Aloe-emodin						
**(6)**	SYD	Unidentified	MKP1/NF-κB/NLRP3	Improves the expression of mucins and occludin	2.25 g/kg, BW	DSS-colitis in mice (5%)	No obvious toxicity to RAW264.7 cells (100 mg/mL) for 24 h	Wei et al. (Wei et al., 2021)
**(7)**	ACB	Fourteen active ingredients, such as Cirsiliol and Astragalin	NF-κB	Restores intestinal barrier proteins and regulates the gut microbiota	50 mg/kg, 100 mg/kg, per day for 7 days	3% DSS-colitis in mice	No obvious toxicity to RAW264.7 cells (1 mg/mL, 24 h)	Dong et al. (Dong et al., 2022a)
**(8)**		Rhein	PI3K/Akt/mTOR	Modulates gut microbiota, changes purine metabolism	50 mg/kg and 100 mg/kg, daily, for 7 days	Four rounds of 2% DSS in mice	Not available	Wu et al. (Wu et al., 2020); Dong et al. (Dong et al., 2022b)
**(9)**	JGQD	Unidentified	Unidentified	Induces *Akkermansia* and *Romboutsia* and suppresses *Escherichia-Shigella*, induces production of propionate and SCFAs, and regulates the metabolism of fatty acids, bile acids, and amino acids	89.96 mg/kg and 179.92 mg/kg, daily, for 7 days by gavage	5% DSS-induced colitis in rats	Not available	Li et al. (Li et al., 2021a)
**(10)**	YFBP	Quercetin, benzoylnapelline, kaempferol, (R)-norcoclaurine, and karakoline	TLR4/NF-κB/NLRP3	Repairs the Intestinal Epithelial Barrier, and Modulating Intestinal Microbiota	4.59 g/kg/d by gavage for 7 days	5% DSS-induced colitis in rats	Not available	Yang et al. (Yang et al., 2023)
**(11)**		Ginsenoside Rg1	Unidentified	Modulates Gut Microbiota and Microbial Tryptophan Metabolism	2 mg/10 g, b.w	DSS-colitis in mice	Not available	Cheng et al. (Cheng et al., 2022)
**(12)**	XLP	Berberine, jatrorrhizine, costunolide, coptisine, and palmatine	PI3K/Akt/mTOR	Promotes the repair of intestinal epithelial cells via blocking PI3K/Akt/mTOR pathway and thus elevating autophagy	0.8, 1.6, 3.2 g/kg, once daily, by gavage, for 7 days	DSS-colitis in mice	Not available	Wang et al. (Wang et al., 2020a)
**(13)**	SLBZS	Ginsenoside Rg1, ginsenoside Re, ginsenoside Rb1, liquiritin, and atractylenolide III	MAPK/NF-κB/pyroptosis	Protects colonic barrier integrity, reduces the production of cytokines	1.183 g/kg, 2.366 g/kg, and 4.732 g/kg	3% DSS-colitis in mice	No cytotoxicity to MCME cells 0.625% in culture medium for 12 h	Chao et al. (Chao et al., 2020)
**(14)**	JGD	Caffeoylquinic acids, and the flavonoid, quercetin-3-O-galactoside	Unidentified	Reduces the levels of mediators associated with oxidative stress	5, 25 and 50 mg/kg bw, p.o	3% DSS-colitis in mice	Not available	Valero et al. (Valero et al., 2020)
**(15)**	KD	Gallic acid, paeoniflorin, emodin, berberine, coptisine, palmatine, jatrorrhizine, baicalein and baicalin	PI3K/AKT/NF-κB	Changes the composition of gut microbiota	1 mL/kg, 2 mL/kg and 10 mL/kg, 15-days by gavage	5% DSS-colitis in rats	Not available	Liu et al. (Liu et al., 2020)
**(16)**	HQD	117 active compounds	Ras-PI3K-Akt-HIF-1α and NF-κB; ESR1; mTOR	Regulates the gut microbiota; relieves endothelial dysfunction; upregulates amino acid metabolism	2.275, 4.55, and 9.1 g/kg, once daily, by gavage, for 7 days	3% DSS-colitis in mice	No obvious toxicity (0–400 μg/mL) to FHC cells for 24 h	Li et al. (Li et al., 2019)
**(17)**	CSD	Matrine, Oxymatrine, Gallic acid, Glycyrrhizic acid, Liquiritin, Ginsenoside Rb1, Indigo, Indirubin and Notoginsenoside R1	Nortch	Enhances the expression of the tight junction proteins, such as ZO-1 and occludin, improves MUC2 production, and reduces the ratio of M1/M2 macrophages	3.64 g/kg, 7.28 g/kg, 14.56 g/kg, once daily, by gavage, for 7 days	DSS-colitis in mice	Not available	Wu et al. (Wu et al., 2021)
**(18)**		Tiliroside	HIF-1α	Regulates aerobic glycolysis of macrophages	2.5, 25.0, and 50.0 mg/kg, by gastric gavage for 7 days	2.5% DSS-induced colitis in mice	Not available	Zhuang et al. (Zhuang et al., 2021)
**(19)**	QCWZD	Twenty-seven compounds	JAK2-STAT3	Reduces proportion of Th17 cells	0.5 g/kg/d, 1.0 g/kg/d, for 7 days, by gavage	DSS-colitis in mice	Not available	Xia et al. (Xia et al., 2022)
**(20)**	WMW	104 active compounds	AGE-RAGE	Preventes the expression of multiple inflammatory mediators	3, 6, 12 g/kg, oral gavage once per day	DSS-colitis in mice (2%, three cycles)	Not available	Duan et al. (Duan et al., 2023)
**(21)**	LWE	Liquiritin and glycyrrhizic acid	TLR4/MyD88/NF-κB	Inhibits colonic inflammation and promoting the levels of tight junction proteins	2, 4, 8 g/kg/d, by gavage for 7 days	3% DSS-colitis in mice	Not available	Shi et al. (Shi et al., 2022)

(1) DMD, dahuang mudan decoction; (2) BPL, *B. pinnatum* leaf; (3) HSW, *Polygoni multiflori* Radix (Heshouwu); (4) SBGP, *Scutellaria baicalensis* georgi polysaccharide; (6) SYD, shaoyao decoction; (7) ACB, anemone chinensis bunge aqueous enema; (9) JGQD, jiawei gegen qinlian decoction; (10) YFBP, yiyi fuzi baijiang powder; (12) XLP, xianglian pill; (13) SLBZS, Shen-Ling-Bai-Zhu-San; (14) JGD, Jasonia glutinosa (L.) DC.; (15) KD, kuijieyuan decoction; (16) HQD, huangqin decoction; (17) CSD, compound sophorae decoction; (19) QCWZD, qingchang wenzhong decoction; (20) WMW, wumei wan; (21) LWE, Glycyrrhiza uralensis Fisch. (Licorice) water exact.

### 3.1 Major mechanisms

#### 3.1.1 Restores tight junction proteins

The tight junctions are junctional protein complexes that regulate the transportation of solutes and water between epithelial cells and also play an essential role in modulating the structure and permeability of epithelium (Zeisel et al., 2019). The tight junctions are mainly composed of three types of membrane-binding proteins, occludin, claudins, and ZO-1. Many previous studies have revealed the role of tight junction proteins in maintaining gut homeostasis. While downregulation of multiple tight junction proteins is associated with the development of IBD(25) and has been found in both UC patients and animals with experimental colitis (Landy et al., 2016; Hu et al., 2021). Some recent studies found that many decoctions/compounds in traditional medicine have a role in ameliorating colonic inflammatory conditions by regulating the expression of tight junction proteins and thereby improving the integrity of intestinal barrier function. An example of this line of evidence was reported by (Huang et al., 2022), showing that Dahuang Mudan decoction (DMD), a widely used prescription for treating intestinal diseases, could significantly intensify the expression of ZO-1, occludin, and Claudin-1. Moreover, using *in vitro* experiments, the researchers verified that DMD could promote the expression of the three tight junction proteins and enhance the migration of Caco-2 cells. Of note, these changes were accompanied by an increase in the proportion of natural cytotoxicity receptor (NCR)+ILC3 and IL-22+ILC3, and decreased ratio of NCR-ILC3. The effect of DMD on tight junction proteins might act as one of the mechanisms that DMD achieved its function in restoring body weight loss, colon shortening, and other clinical conditions induced by DSS.

The role of traditional medicine in regulating tight junction proteins was also evidenced by many other studies, such as the study by (Andrade et al., 2020), showing that the anti-UC effects of *Bryophyllum pinnatum* (Lamarck) Leaf (BPL), a medicinal plant widely used in traditional medicine in Brazil for treating inflammatory diseases, was at least partially due to its regulation of ZO-1. The study by (He et al., 2021) showed that the major bioactive constituent in water extract of *Polygoni multiflori* Radix (Heshouwu, HSW), 2,3,5,4′-tetrahydroxystilbene-2-O-β-D-glucoside (TSG), could improve the expression of tight junction proteins, such as ZO-1 and occludin, thus restore intestinal epithelial barrier structure. Cui et al. (2021) isolated a homogeneous polysaccharide from *Scutellaria baicalensis* Georgi (SBG), named SP2-1, and studied its roles in DSS-induced colitis. SP2-1 treatment reduced colonic pathological damages, and increased the expression of tight junction proteins, including ZO-1, occludin, and claudin-5, and thus improved the intestinal integrity of the colonic barrier.

Additionally, some alternative therapies also had a role in restoring tight junction proteins. For instance, the combination of moxibustion with acupuncture could enhance the levels of tight junction proteins in Crohn’s disease patients (Shang et al., 2015). Targeting the tight junction proteins is also a mechanism employed by some drugs for the clinical treatment of IBD, such as the JAK inhibitor, tofacitinib, which could normalize the expression level of claudin-2 and occludin (Spalinger et al., 2021). Another medicine used in treating IBD possibly by regulating tight junction proteins is infliximab, a monoclonal antibody targeting TNF-α. The blockade of TNF-α had a role in relieving inflammation-induced blood-retinal barrier by restoring the expression of claudin-1 and occludin in a diabetic rat model (Xie et al., 2017). This effect of infliximab might contribute to its role in reducing enterocyte apoptosis and thus restoring the colonic barrier in experimental colitis (Yakymenko et al., 2018). A direct effect of infliximab on the expression of the tight junction proteins in the colitis model was provided by a study by (Xiao et al., 2022) showing that administration of infliximab significantly improved the level of occludin but did not affect ZO-1 expression in DSS-induced colitis model. These studies further demonstrated that regulating the expression of tight junction proteins is a promising strategy for treating colonic damage in IBD. However, the chemical ingredients and their specific roles of some traditional decoctions or naturally occurring compounds have not been determined, further studies that address these issues may facilitate the development of new drugs for treating UC via regulating the tight junction proteins.

#### 3.1.2 Induces mucins

Mucins are a family of heavily glycosylated hydrophilic proteins produced by epithelial goblet cells that serve as the major component of mucus, which forms a physical barrier lining the intestine (Peterson and Artis, 2014) and plays multiple roles in the protection of mucosal surfaces, such as the defense function against invading bacteria (Frenkel and Ribbeck, 2015). Studies have shown that dysregulation of mucins is closely associated with the development of IBD (Niv, 2016; Cantero-Recasens et al., 2022). Muc2 deficiency causes spontaneous colitis in mice, indicating a prominent role in mucins in maintaining gut immune homeostasis (Wenzel et al., 2014). Moreover, the role of mucins in IBD is further proved by a recent study by Al-Shaibi et al. (2021) showing that human deficiency of anterior gradient 2 (AGR2), a disulfide isomerase involved in mucin processing, causes mucus barrier dysfunction and results in infantile IBD. Therefore, restoration of mucin production may be a promising manner for treating some symptoms of IBD.

A study by Shi et al. (2021) found that the effect of Aloe vera on colitis had a relation to its role in regulating mucus production. Aloe vera is an herbal plant widely used in traditional Chinese medicine for treating intestinal constipation and colitis. The researcher disclosed that Aloe vera extract could significantly decrease DSS-induced clinical symptoms. At the same time, it upregulated the production of mucin proteins, such as MUC2 and MUC5AC, and thus enhanced the thickness of the colonic mucus layer. Moreover, in an *in vitro* experiment, the researchers revealed that the mucin-promoting role of Aloe vera was mediated by its major constituent, aloin A, which increased the level of MUC2 in LPS-stimulated LS174T cells. Another example that may demonstrate the role of mucin in colitis was reported by Wei et al. (2021), showing that one mechanism of Shaoyao decoction (SYD), a traditional Chinese medicine that has shown effects in treating UC, in alleviating colitis was its function in enhancing mucin production and thereby strengthening the integrity of the intestinal barrier. Moreover, (Dong et al., 2022a) investigated the effect of Anemone chinensis Bunge (ACB) aqueous enema on colonic inflammation. ACB is a medicinal herb that has been used as an ingredient in enema to relieve UC-associated symptoms. The researchers found that the major compounds in ACB might improve the expression of mucin proteins, including mucin-2 and mucin-3A. In addition, (Andrade et al., 2020), found that the anti-UC effects of BPL extract were related to its role in promoting MUC-3 expression. Of note, regulating the expression of mucin is also a critical mechanism employed by some existing medicines or dietary supplies in treating IBD. For example, (Roman et al., 2017), showed that infliximab treatment could significantly recover mucus production and thus contribute to the amelioration of DSS-induced colonic inflammation in rats. A study by Faure et al. (2006) found that a dietary supply of specific amino acids, such as threonine, serine, and proline, could promote mucin synthesis and thus improve mucosal healing in DSS-treated rats. Thus, enhancing mucin production is an effective strategy that contributes to the remission of some UC-associated symptoms. While the bioactive compounds in some decoctions, such as SYD and ACB need to be further identified, which may improve the effect of the decoctions in treating UC since some unrelative constituents may cause reverse effects in the inflamed colon.

#### 3.1.3 Regulates microbiota and associated metabolites

The gut microbiota is the microorganisms living in the gastrointestinal tract, mainly including bacteria, fungi, viruses, and protozoa. The normal structure and composition of the microbiota may act as a part of the intestinal barrier and play a prominent role in maintaining gut homeostasis (Nishida et al., 2018). While its dysregulation is associated with many human diseases, including UC(43). The gut microbiota may release some metabolites, such as bile acids and short-chain fatty acids (SCFA) (Liu et al., 2022), which serve as the nutrients of some bacteria and also have a role in regulating the composition of the microbiota. More importantly, the metabolites may regulate immune maturation and barrier integrity and in particular environments, some metabolites, such as SCFA, may trigger the development of IBD (Zheng et al., 2022). DSS treatment changes the composition of gut microbiota by increasing the genus level of Lachnospiraceae_NK4A136, while suppressing the level of *Helicobacter*, *Bacteroides*, and Parabacteroides (He et al., 2021). Studies have shown that the gut microbiota and their metabolites are targets of some decoctions or natural compounds that affect the progression of disease in UC models. For example, in a study by He et al. (2021), the researchers found that TSG, a bioactive component of PMR, could enhance the abundances of Firmicutes and Bacteroidetes to improve the composition of gut microbiota, which was an important mechanism of TSG to achieve its anti-UC effects. Another compound that may affect the progression of colitis via regulating gut microbiota is Rhein reported by Wu et al. (2020). Rhein is a major bioactive component of the herbal plant rhubarb. Using an experimental colitis model, the researchers found that this compound was efficacy in alleviating inflammatory damages induced by DSS. In the examination of metabolic profiles of the colon tissues using non-targeted metabolomics, they revealed that uric acid was a critical regulator of colonic inflammation. Rhein treatment could significantly reduce the level of uric acid, which plays a detrimental role in the integrity of the intestinal barrier. Furthermore, Rhein might change purine metabolism and induce an alteration in the composition of the gut microbiota, which was sufficient to relieve DSS-induced colonic damage. Thus, Rhein and its role in regulating gut microbiota may act as a new approach to treating colitis.

The effect of traditional medicine on colitis via targeting microbiota was also proved by other studies of traditional decoctions, such as the one reported by the study by Li Q. et al. (2021). In this study, investigated the potential effect of Jiawei Gegen Qinlian Decoction (JGQD) on gut microbiota of rat with colitis under different dietary environments. JGQD is an effective formula used in treating acute enteritis diarrhea for centuries. The researchers showed that JGQD administration induced a relief of colon inflammation in the mice, which manifested reduced body weight loss, bloody stool, diarrhea colon shortening, and DAI. The researchers then highlighted the importance of gut microflora in the anti-UC effect of JGQD using a depletion study, in which they treated the animals with antibiotics and found that JGQD lost its protective role to DSS-treated rats. This finding confirmed the role of microflora in the function of JGQD. Moreover, this study also revealed that JGQD could induce an increase in *Akkermansia* and *Romboutsia* and a decrease in *Escherichia-Shigella*. These alterations together with changes in metabolites resulted in the remission of colonic inflammation via many mechanisms, such as expanding the preexisting microbe-specific Tregs (Kuczma et al., 2020). Thus, the anti-UC function of JGQD were largely dependent on gut microbiota and might have the potential to application as a complementary medicine in UC treatment. Another study by Yang et al. (2023) reported that Yiyi Fuzi Baijiang Powder (YFBP), a classic Chinese herbal formula commonly used in treating UC, might regulate the diversity and richness of intestinal microbiota and thus affect the severity of experimental colitis using a strategy combined with network pharmacology, screening, and bioinformatics.

Alterations in gut microbiota-related metabolites also affect the development of UC-associated colitis. Cheng et al. (2022) reported that the natural compound, ginsenoside Rg1, could alleviate the colon damage and inflammation inflammatory conditions induced by DSS in mice. It also protected the colon barrier function by improving the dysregulated composition of gut microbiota. This function was achieved by regulating several metabolic pathways, such as the metabolism of tryptophan, vitamin B6, and valine. Moreover, in the study investigating the role of JGQD in colitis by Li Q. et al. (2021), they found that the effect of JGQD might be associated with its role in inducing the production of propionate and total short-chain fatty acids (SCFAs) and regulating the metabolism of fatty acids, bile acids, and amino acids in the colon. Cui et al. (2021) studied the polysaccharide isolated from SBG (SP2-1) in DSS-induced colitis. The researcher found that SP2-1 treatment enhanced particular metabolites of gut microbiota, including acetic acid, propionic acid, and butyric acid. Along with these changes, the composition of microflora was also changed by administration of SP2-1, which significantly elevated the abundance of *Firmicutes*, *Bifidobacterium*, *Lactobacillus*, and *Roseburia*, and inhibited the levels of *Bacteroides*, *Proteobacteria* and *Staphylococcus* in the gut.

It should be noted that regulating the structure of microbiota is also a mechanism of existing UC-treating drugs in healing colonic damage. Although the direct relation between infliximab and microbiota has not been revealed, it has been shown that infliximab could change the composition of gut microbiota (Seong et al., 2020), possibly by blocking the effect of TNF-α in inducing antimicrobial peptides (Morio et al., 2023), which play an important role in regulating the composition of gut microbiota (Gubatan et al., 2021). Another drug used in IBD treatment, 5-aminosalicylic acid (5-ASA), was also reported to have a role in altering fecal bacterial microbiota in patients with UC (Xu et al., 2018). Therefore, targeting gut microbiota and the related metabolites by decoctions in traditional Chinese medicine represents a new treatment strategy for UC patients. However, the changes in microflora by administration of some decoctions may be dependent on the combination of numerous compounds. It is hard work to clarify if some compounds are available that may promote the proliferation of bacteria harmful to the host. Future studies may focus on the development of strategies that can identify these compounds and subsequently reduce the proportion of these compounds in particular decoctions.

#### 3.1.4 Suppresses the infiltration of inflammatory cells and mediators

The maintenance of the intestinal epithelial barrier requires a balance of pro- and anti-inflammatory mediators in the gut (Barbara et al., 2021). An inflammatory microenvironment predisposes the epithelial barrier to the invading pathogens via increasing permeability of the epithelium, thus the immune cells in the blood may infiltrate into the mucosa and produce various inflammatory mediators, which further amplify the immune response in the gut (Luissint et al., 2016). Thus, inhibiting immune cell infiltration and inflammatory mediators and thereby restoring the intestinal barrier integrity is an effective strategy for treating UC, as evidenced by the application of many clinically used medicine for UC patients, such as Vedolizumab, an α4β7 integrin antagonists that can block lymphocytes migration to the gastrointestinal tract (Cherry et al., 2015), and 5-ASA, an anti-inflammatory drug that inhibits the productions of pro-inflammatory cytokines and mediators (Mehta et al., 2023). Recent studies have proved that some traditional medicine could suppress the progression of UC models by inhibiting inflammatory cell infiltration and the production of pro-inflammatory cytokines. In this line of evidence, Cui et al. (2021) found that the polysaccharide SP2-1 could reduce colonic pathological damage, and meanwhile, suppressed the levels of pro-inflammatory cytokines. Furthermore, the study by He et al. (2021) showed that the major bioactive constituent in water extract of PMR, TSG, could suppress the production of pro-inflammatory cytokines, including TNF-α, IL-1β, and IL-6, and enhance the level of anti-inflammatory cytokine IL-10. Similarly, as reported by Shi et al.‘s study (Shi et al., 2021), the expression of colonic pro-inflammatory mediators, including IL-6, IL-1β, and TNF-α were suppressed, yet the expression of IL-10 was upregulated by Aloe vera treatment, suggesting a role of this herb in regulating inflammatory cytokines. In addition, in the investigation of the effect of Xianglian Pill (XLP) on colitis, (Wang et al., 2020a), found that XLP exposure could suppress the levels of pro-inflammatory cytokines in the colon. XLP is a traditional Chinese medicine composed of *Coptidis Rhizoma* and *Aucklandiae Radix* that has been used in treating intestinal diseases. Its anti-UC effect was associated with its inhibition of NF-κB and PI3K signaling pathways.

In a study by Chao et al. (2020), the researchers investigated the effect of Shen-Ling-Bai-Zhu-San (SLBZS), a formula in traditional Chinese medicine used in the management of many gastrointestinal diseases, in a colitis model induced by DSS. They found that SLBZS could improve DSS-induced body weight loss, and colonic damage, and decrease the DAI score by inhibiting the production of pro-inflammatory cytokines, including IL-1β, IL-18, and TNF-α, in both colon tissues and colonic epithelial cells. Valero et al. (2020) studied the effect of *Jasonia glutinosa* (L.) DC. (JGD), a traditional herbal medicine also known as rock tea (RT), on colonic inflammation induced by DSS and found that RT had a significant role in suppressing DSS-induced pathological changes and showed a protective role for the intestinal barrier function. The major constituents of RT, including phenolic compounds and pigments, could reduce the production of pro-inflammatory cytokines, such as TNF-α and IL-6. In addition, in the study by Andrade et al. (2020), the researchers explored the role of BPL extract in experimental colitis. BPL extract induced a decline in clinical symptoms. This effect was accompanied by a reduction in cell adhesion molecules, which was associated with the decrease of colonic infiltrating leukocytes. All these changes may regulate the intestinal barrier function and affect the disease progression. Thus, the anti-UC activity of various traditional medicine was related to their roles in inhibiting pro-inflammatory cytokines and related immune cell infiltration to the mucosa.

#### 3.1.5 Reduces oxidative stress in the gut

Oxidative stress is a condition caused by an imbalance between the production and elimination of reactive oxygen species (ROS). It can be induced by various environmental factors, such as radiation and antibiotics, that disrupt the ROS balance (Pizzino et al., 2017). Previous studies have revealed that oxidative stress may disrupt the intestinal epithelial and endothelial tight junctions via regulating protein modification, such as thiol oxidation and phosphorylation (Rao, 2008). Thus, suppression of oxidative stress significantly contributes to the restoration of the damaged intestinal barrier (Jena et al., 2012; Pereira et al., 2015). As mentioned above, in Valero et al.’s study (Valero et al., 2020) about the effects of RT on colonic inflammation, the authors found that RT had a significant role in reducing the levels of mediators associated with oxidative stress, such as superoxide radicals, Arachidonate 5-lipoxygenase (5-LOX), iNOS, NO, and COX2. Thus, the anti-UC activity of RT was closely related to its antioxidant activities.

In agreement with this point, (Andrade et al., 2020), studied the anti-oxidant role of BPL extract in both 2.4-dinitrobenzene sulfonic acid (DNBS) and DSS-induced colitis models. They found that BPL extract induced could inhibit the activity of NF-κB, leading to a decrease of pro-inflammatory mediators and chemokines. The alteration in NF-κB activity may have crosstalk with the oxidative stress in the colon (Morgan and Liu, 2011), as evidenced by the decreased activity of malondialdehyde and myeloperoxidase and increased level of glutathione after treatment with BPL extract. Moreover, (Liu et al., 2020), studied the effect of Kuijieyuan Decoction (KD) on intestinal barrier integrity in the colitis model. KD is a traditional Chinese medicine widely used to alleviate UC-associated intestinal injury. The researchers found that KD treatment significantly alleviated UC injury via suppressing oxidative stress, as evidenced by inhibited levels of malondialdehyde (MDA), a marker of oxidative stress. KD increased the production of anti-oxidative molecules, including SOD, GPx, and CAT. Thus, the role of KD in improving intestinal barrier function was largely associated with its effect on altering oxidative stress, which was mediated by the TLR-induced PI3K/AKT/NF-κB pathway. Together, these studies indicated that inhibition of oxidative stress is a prominent strategy employed by many traditional medicinal decoctions for suppressing UC-related clinical changes. Some previous studies have revealed that some drugs used in UC treatment also affect oxidative stress in the colonic tissue. For instance, a study by Caltabiano et al. (2011) reported that the administration of 5-ASA could reduce the levels of oxidative DNA in colonic tissues. Another study by Peng et al. (2020) showed that 5-ASA had a role in alleviating manganese-induced oxidative damage by inhibiting ROS production. It should be noted that some drugs used in UC treatment may enhance oxidative stress in the colon. An example of this line of evidence is provided by glucocorticoids, a type of powerful anti-inflammatory and immunomodulatory agent used to treat many inflammatory diseases, including IBD (Bruscoli et al., 2021). Some early studies had observed that long-term treatment of glucocorticoids could induce oxidative stress (Bjelakovic et al., 2007), which may partially explain the adverse effects occurring in glucocorticoid treatment (Oray et al., 2016). These studies suggest that appropriate management of oxidative stress is an important issue that needs to be considered during the treatment of UC.

### 3.2 Related signaling pathways

#### 3.2.1 NF-κB/MAPK

NF-κB is a critical transcription factor involved in many biological processes and its overactivation may disrupt the intestinal barrier via various mechanisms and thus affect the development of IBD, including UC (Baker et al., 2011; Zhang et al., 2017). It is reasonable to assume that the NF-κB signaling pathway is the most commonly affected target of many herbal decoctions or natural compounds based on the analysis of recent studies. For instance, (Andrade et al., 2020), studied the anti-inflammatory role of BPL extract in experimental colitis. BPL extract induced a decrease in disease severity by inhibiting the levels of TLRs and NF-κB p65, leading to a decrease of pro-inflammatory mediators and chemokines, suggesting that BPL extract might exert the anti-inflammatory effects via regulating NF-κB. Another piece of evidence that may prove NF-κB to be the target of traditional medicinal decoctions is provided by the study of (He et al., 2021), showing that TSG could suppress the production of pro-inflammatory cytokines, such as TNF-α, IL-1β, and IL-6, and enhance the level of anti-inflammatory cytokine IL-10, indicated a potential effect of this compound on suppressing the NF-κB signaling pathway since the production of pro-inflammatory cytokines tested in this study is all dependent on the activation of NF-κB. Similarly, in a study by Shi et al. (2021), the expression of colonic pro-inflammatory mediators, including IL-6, IL-1β, and TNF-α, were suppressed, yet the expression of IL-10 was upregulated by Aloe vera treatment, suggesting a role this herb in regulating the NF-κB signaling pathway. However, in most of these studies, the exact target of the herbal medicine or compounds in the NF-κB pathway has not been determined. As mentioned above, BPL may affect this pathway by suppressing the expression of the receptor protein TLRs, while for some other compounds or herbal medicine, some intracellular molecules in this pathway, such as p65, can be affected. The identification of the target molecules of a particular medicine in the NF-κB signaling pathway may be helpful for reducing toxicity of the medicine in treating UC. While it is also possible that NF-κB is only one of the pathways that affected by a particular medicine, the anti-UC effect of the medicine may be dependent on suppression of multiple pathways. Thus, a comprehensive analysis for the alterations in gene expression and activation induced by a compound or herbal medicine is important for understanding their roles in treating UC.

#### 3.2.2 PI3K-AKT

PI3K-AKT pathway is an intracellular signaling pathway critical for many biological processes, such as cell survival, proliferation and angiogenesis (Shao et al., 2017). The activation of this pathway contributes to the disruption of intestinal barrier function (Shao et al., 2017). Many decoctions in traditional medicine could regulate the intestinal barrier and thus have a role in alleviating UC associated symptoms via regulating the PI3K-AKT pathway. An example in this line of evidence was provided by Wang et al. (2020b), who studied the effect of Xianglian Pill (XLP) on intestinal inflammation in a DSS-induced colitis model in mice. As a traditional Chinese medicine composed of *Coptidis Rhizoma* and *Aucklandiae Radix,* XLP has been used in treating intestinal diseases. Using an integrative pharmacology-based approach, the researchers found that XLP could protect the colon epithelial barrier by promoting the repair of intestinal epithelial cells via blocking the PI3K/Akt/mTOR pathway and thus elevating autophagy. It should be noted that PI3K/Akt/mTOR pathway was also a target of Rhein to exert the anti-colitis role in regulating the level of uric acid (Dong et al., 2022b). Furthermore, Shi et al. (2021) found that the role of Aloe vera in promoting mucin production was mediated by up-regulating p-PKC and p-ERK and downregulating p-PI3K and p-AKT. Similar to this study, (Li et al., 2019), found that HQD had an impact on Ras-PI3K-Akt-HIF-1α pathway, thus regulating the composition of gut microbiota in a DSS-induced colitis model. The effect of HQD was similar to mesalazine, a medicine belonging to aminosalicylates used in treating UC (Kato et al., 2018). Additionally, as mentioned above, in the study of (Liu et al., 2020), the researchers studied the effect of KD on intestinal barrier integrity in colitis and found that KD had a role in alleviating UC symptoms. Its inhibitory role for oxidative stress was associated with its effect on downstream signaling pathways, including p-PI3K, p-AKT, and p-NF-κB. Thus, the activation of these pathways was a critical mechanism of KD in improving intestinal barrier function. Based on the analysis of these studies, it is observed that PI3K-AKT is a common signaling pathway employed by many traditional medicines to achieve their anti-UC functions. Besides the regulation of the PI3K-AKT pathway, the activation or suppression of other related molecules, such as mTOR and HIF-1α, as described later in this paper, may play a more important role in recovering the intestinal barrier by various traditional medicines. While the ingredients in these decoctions need to be identified and their potential toxicity and safety in animal models need to be further evaluated before preclinical applications.

#### 3.2.3 Pyroptosis

Pyroptosis is a type of cell death that is induced by inflammatory signals and related to the activation of inflammasomes (He et al., 2015). Some stimulants are sensed by inflammasome-forming sensors, which then trigger the activation of caspase-1. The latter induces the cleavage of GSDMD and releases the active N-terminal protein, which then locates to the plasma membrane and forms pores to initiate cell lysis (He et al., 2015). Pyroptosis of intestinal epithelial cells plays an important role in barrier dysfunction and gut inflammation (Zhang et al., 2022). Thus, targeting pyroptosis has been proved to be an effective way to treat UC in animal models. An example of pyroptosis as a target of traditional medicine in protecting the intestinal barrier is the study of Shen-Ling-Bai-Zhu-San (SLBZS), a formula in traditional Chinese medicine used in the management of many gastrointestinal diseases. In this study, (Chao et al., 2020), showed that the effects of SLBZS on colon inflammation were mediated by its inhibition of pyroptosis related genes, including the levels of NLRP3, ASC, and GSDMD-N, and the activation of caspase-1 and caspase-11 in the colons. The researchers also found that the expression of most of these genes was also affected by MAPK and NF-κB signaling pathways. Reduced pytoptosis of colonic epithelial cells might be an explanation for the increased levels of colon tight junction proteins, such as ZO-1 and occludin, which increased colonic barrier integrity and alleviated the severity of colon inflammation induced by DSS. Thus, the anti-UC effect of SLBZS was achieved by suppressing MAPK/NF-κB signaling pathway and pyroptosis, thereby enhancing the production of tight junction proteins.

Another formula that could protect the intestinal barrier by regulating pyroptosis is Shaoyao decoction (SYD), a traditional Chinese medicine that has shown effects in treating UC. A report by Wei et al. (2021) studied its role in colitis and found that SYD could significantly reduce the inflammatory index and improve the expression of mucins and occludin. Regarding the mechanisms, SYD could improve the integrity of colon barrier function by suppressing the level of pyroptosis, as evidenced by the reduction of caspase-1 activity and the release of GSDMD, thus suppressing the expression and activation of the NLRP3 inflammasome. Moreover, SYD showed an effect in suppressing the level of necrosis of the colon tissues revealed by TUNEL staining. Further studies revealed that the effect of SYD was attributed to its role in activating mitogen-activated protein kinase phosphatase-1 (MKP1) since MKP1 inhibitor would suppress the protective role of SYD on colonic changes induced by DSS. This study thus indicated the role of MKP1 in the regulation of the NLRP3 inflammasome and pyroptosis. In addition, as shown by Yang et al. (2023), Yiyi Fuzi Baijiang Powder (YFBP) could also be a decoction that affects the progression of colitis via regulating the NLRP3/pyroptosis pathway. YFBP is a classic Chinese herbal formula commonly used in treating UC. Using a strategy combined with network pharmacology, screening, and bioinformatics, the researchers revealed that the inhibition of intestinal pathological changes by YFBP was related to its role in preventing the activation of the NLRP3 inflammasome and pyroptosis, which led to an increase of tight junction proteins in the colons and a change of gut microbiota. Thus, traditional medicine may affect the activation of NLRP3 inflammasome and pyroptosis is largely dependent on its regulation of some upstream regulators, such as MAPK/NF-κB and MKP1, and thus affects the activation of inflammasome and inflammasome-associated pyroptosis. However, the compounds with anti-pyroptosis activities in traditional medicine mentioned above have not been identified. The exact targets of these compounds in pyroptosis-associated signaling pathways and their potential toxicities also need to be determined in future studies.

#### 3.2.4 Notch

The Notch signaling pathway is a highly conserved pathway that modulates the stem cell fate and phenotype in the intestinal mucosa and thus plays a role in the maintenance of intestinal barrier function (Fazio and Ricciardiello, 2016). It has been shown that regulating the Notch pathway may inhibit intestinal inflammation and restore the integrity of the intestinal barrier (Fazio and Ricciardiello, 2016; Wu et al., 2021) studied the role of compound sophorae decoction (CSD), an herbal formula that has been used clinically in patients with UC, in DSS-induced colitis model in mice and found that CSD administration can markedly reduce the clinical manifestations and pathological changes. For the mechanisms, the decoction enhanced intestinal barrier function by reducing apoptosis of epithelial cells and thus facilitating epithelial cell regeneration. Moreover, CSD treatment significantly enhanced the expression of the tight junction proteins, such as ZO-1 and occludin, improved MUC2 production, and reduced the ratio of M1/M2 macrophages. It should be noted that these changes induced by CSD were associated with reduced levels of Notch1 and Hes1 and increased ATOH1 and MUC2, indicating a decreased activity of the Notch signaling pathway. It should be noted that the Notch signaling pathway is one of the major pathways involved in the regulation of intestinal barrier function (Fazio and Ricciardiello, 2016; Ghorbaninejad et al., 2019). Its appropriate regulation and interplay with other signaling pathways, such as the NF-κB and PI3K-AKT pathways, are important in regulating the production of barrier-protecting proteins, including the tight junction proteins and mucins. Similar to other decoctions, the relative contributions of CSD ingredients to the inhibitory effects of this medicine on the Notch signaling pathway and other related pathways have not been evaluated. Meanwhile, the lack of data about the safety evaluation and toxicity estimation of CSD may limit its further development and application in UC treatment.

#### 3.2.5 HIF-1α

HIF-1α is a crucial regulator of oxygen homeostasis. It is essential for immunological responses and plays a critical role in the maintenance of gut homeostasis and the integrity of intestinal barrier function. Impaired regulation of HIF-1α is associated with the development of ulcerative colitis (Yin et al., 2022). Some studies have shown that modulating the activity of HIF-1α using traditional medicine could ameliorate colitis. For instance, a report by Zhuang et al. (2021) studied the role of Tiliroside, a bioactive compound with anti-inflammatory and anti-oxidant activities extracted from several dietary plants, in an animal model of colitis and found that tiliroside could alleviate clinical symptoms of colitis and improve pathological damages in the colon in a macrophage dependent manner. The compound increased the proportion of M2 macrophages. For the mechanisms, the researchers showed that the tiliroside had a role in regulating the aerobic glycolysis of macrophages. Most importantly, the tiliroside could suppress proteasomal degradation of HIF-1α and thereby downregulate HIF-1α-induced enzymes related to glycolysis. The anti-colitis role of tiliroside might be achieved by regulating HIF-1α modulated glycolysis and thus inducing macrophage reprogramming in the colon. Although the authors claimed that tiliroside had minimal effects on epithelial barrier function, while a previous study by Shao et al. (2018) disclosed that deficiency of HIF-1α in the intestine may disrupt the barrier function. HIF-1α deficient mice display reduced production of epithelial tight junction proteins, including claudin-1 and occludin. Therefore, further studies are needed to confirm the impact of HIF-1α on intestinal barrier function and its role in the regulation of tight junction proteins.

#### 3.2.6 RORγT

RORγT is a transcription factor that controls Th17 cell differentiation and is crucial for the secretion of Th17 effector cytokines (Kumar et al., 2021). Previous studies have shown that Th17 cells may infiltrate into the inflamed intestine of UC patients and produce effector cytokines, such as IL17A, to amplify the inflammatory process in the gut. Regulating RORγT production and thus keeping the activity of Th17 cells in check are effective for the management of many inflammatory diseases, including IBD (Galvez, 2014). Some recent studies have also shown that decoctions of traditional medicine have a role in regulating RORγT activity and thereby affecting the progression of UC. An example of decoction is Qingchang Wenzhong Decoction (QCWZD), a prescription in Chinese medicine used in UC treatment with an unknown mechanism. Xia et al. (2022) studied the role of QCWZD in the colitis model induced by DSS and found that treatment of QCWZD significantly reduced the levels of myeloperoxidase (MPO), inflammatory cell infiltration to the colon and the levels of inflammatory mediators in serum, meanwhile, it promoted expression of occludin and ZO-1. In an attempt to study the mechanisms of QCWZD in suppressing colitis conditions, the researchers showed that the drug could reduce the proportion of Th17 cells in the colon by inhibiting the expression of RORγT. Further studies showed that the reduction of RORγT might be associated with the decreased phosphorylation of the JAK2-STAT3 pathway.

Another decoction that may affect RORγT is Glycyrrhiza uralensis, Fisch. (Licorice). It is a widely used herbal plant in TCM for reducing toxicity and increasing the treating efficacy of drugs. Studies have shown that the major constituents of Licorice have anti-inflammatory roles. Shi et al. (2022) investigated the role of Licorice water extracts (LWE) in DSS-induced colitis and found that LWE had an anti-inflammatory effect via regulating the expression of TLR4/MyD88/NF-κB pathway and promoting the levels of tight junction proteins in the colons, thus improving the colon mucosal barrier integrity. Moreover, LWE showed a role in regulating the imbalance of Th17/Treg proportion induced by DSS administration via influencing the expression of transcription factors ROR-γt and Foxp3. The researcher’s data also revealed a role of LWE in modulating the diversity of gut microbiota. Thus, the roles of traditional medicine in regulating ROR-γt expression are associated with the alterations of some other signaling pathways, such as the JAK2-STAT3 pathway and the NF-κB pathway. ROR-γt may act as one of the effector molecules targeted by various compounds in a decoction in regulating intestinal barrier function. It is important to identify the bioactive ingredients of QCWZD and LWE that target ROR-γt associated signaling pathways and contribute to alleviation of UC related pathological changes in future studies.

#### 3.2.7 AGE-RAGE pathway

The advanced glycation end products-receptor of advanced glycation end products (AGE-RAGE) signaling pathway triggers activation of multiple intracellular pathways (Body-Malapel et al., 2019). Activation of this pathway promotes the production of ROS and enhances oxidative stress, and thus plays an important role in local inflammatory processes in intestinal diseases such as IBD (Body-Malapel et al., 2019; Duan et al., 2023) studied the role of regulating the AGE-RAGE pathway in UC in their study of Wumei Wan (WMW), a traditional decoction used in treating digestive disorders. The researchers found that WMW may affect the development of colonic inflammation mainly by the activation of the AGE-RAGE signaling pathway via several bioactive ingredients, including taxifolin, rutaecarpine, kaempferol, quercetin, and luteolin. More importantly, the researchers validated the role of WMW in regulating AGE-RAGE pathway in DSS-induced colitis model and found that WMW can suppress the progression of colitis and prevented the expression of multiple inflammatory mediators. Meanwhile, it inhibited the production of many genes involved in the regulation of angiogenesis, such as Spp1 and Serpine1 (Rowe et al., 2014; Teng et al., 2021). Moreover, WMW had a role in suppressing RAGE expression and diminishing DSS-induced epithelial damages. However, the mechanisms of WMW in regulating RAGE expression have not been determined. It is possible that some bioactive components of WMW may interact with the promotor region or related transcription factors and thus block the transcription of RAGE. Other possible mechanisms that can be employed by WMW components to regulate AGE-RAGE signaling activity may include the blockade of the interplay between AGE and RAGE, inhibition of the expression and/or activation of RAGE downstream molecules, and RAGE posttranslational modification and degradation. Moreover, the effect of WMW in modulating epithelial damage may indicate a mechanism of this medicine in protecting intestinal barrier, a point that deserve further investigation. Therefore, the AGE-RAGE pathway may be a promising target for UC treatment, but the exact mechanisms by which WMW exerts the anti-UC activity and regulates intestinal barrier need to be extensively studied in future.

## 4 Limitations and possible solutions

As mentioned above, many decoctions in traditional medicine showed protective effects on chemical induced UC models. An important issue that limits the application of some decoctions or compounds is their solubility and bioavailability in inflammatory sites. For instance, as one of the major components of rhubarb, Rhein, has been used in treating many diseases because of its anti-inflammatory activity. While the poor water solubility and low oral bioavailability of Rhein have largely limited its application (Li et al., 2021b). A possible solution for this problem is reported by Wang et al. (2020b), showing that linkage of β-cyclodextrin significantly improved the water solubility of Rhein. Another approach for improving Rhein bioavailability is provided by studies of (Yao et al., 2021). In this study, the researchers conjugated Rhein with deoxycholic acid-functionalized nanoparticles and found that this treatment effectively enhanced the water solubility of Rhein. The third possible approach that may improve the bioavailability of drugs is combined application of adjuvant compounds, such as the supportive role of piperine to solubility of curcumin (Heidari et al., 2023). In addition, some studies have shown that the bioavailability of compounds can be significantly enhanced by delivery-aid carriers, such as lipid nanoparticles and liposomes, which have been reported to have a role in enhancing curcumin delivery to the brain of mice (Yang et al., 2021).

Another issue that needs to be considered before the application of traditional medicine is the efficacy and safety of the ingredients. Some of the decoctions/compounds have been applied for centuries. Thus, their safety has been verified in large amounts of clinical cases. However, the applications may be largely dependent on the clinician’s experience, which may limit the application of same decoction by different clinicians to different patients. The condition may explain the frequently occurred adverse events in application of particular traditional medicine (Wang et al., 2010; Wu et al., 2010; Hwang et al., 2018). Moreover, the bioactive ingredients of the decoctions have not been fully identified and studied in independent experiments. The effects of decoctions may be associated with a combination of various bioactive ingredients, thus the relative ratio of compounds in an herbal plant can be influenced by its growing environment. In addition, the safety concentration of compounds also needs to be evaluated prior to the application. Thus, the role of compounds in decoctions, the effective compound combinations, and the potential toxicities all need to be determined in treating UC in future investigations.

Most of the compounds described in this review are effective to reduce inflammation in animal models of colitis, while their further clinical application may meet other challenges. For instance, the doses on an mg/kg basis that were effective and safe in animal models may be not work for human individuals with UC after scaling of the doses based on body weight. This phenomenon can be possibly attributed to the varied pharmacokinetics of different species (Nair and Jacob, 2016; Nair et al., 2018). For this purpose, a guideline has been developed by U.S. Food and Drug Administration to estimate the human equivalent dose for a compound, which can work as the maximum recommended starting dose (MRSD) for an initial clinical trial (https://www.fda.gov/regulatory-information/search-fda-guidance-documents/estimating-maximum-safe-sta). While before the estimation of MRSD, the bioavailability, major metabolites, and plasma level of the compound need to be determined. However, in most of the currently available studies, the *in vivo* data do not contain sufficient information to estimate the MRSD of the compound. Moreover, most studies do not provide *in vivo* toxicity data that are needed for the estimation of MRSD. Therefore, extensive *in vitro* and *in vivo* studies are required to determine the toxicology, tolerability, and pharmacokinetic profiles. Based on these data, a clinical trial can be designed to evaluate the possible application of a compound in treating patients with UC.

## 5 Conclusion and future prospects

Based on a critical reading and a systemic analysis of recent studies as to the effects of traditional medicine on intestinal barrier function in literature, numerous decoctions and natural compounds have shown a role in animal models of UC by improving the intestinal barrier function via various mechanisms, mainly including regulating the expression of barrier protecting proteins such as the tight junction proteins and mucins, suppressing inflammatory cell infiltration and associated pro-inflammatory mediators, inhibiting oxidative stress, and modulating the composition of gut microbiota and their metabolites. Our further analysis revealed that these intestinal protecting mechanisms may be activated by numerous signaling pathways or active molecules. In this study, we give a few examples of these signaling pathways, mainly including the NF-κB/MAPK, PI3K-AKT, NLRP3 inflammasome/Pyroptosis, Notch, HIF-1α, RORγT, and AGE-RAGE signaling pathways, to elaborate the possible routes employed by traditional medicine in achieving their intestinal protection roles. Of note, there should be other mechanisms that we have not mentioned in this paper be employed by traditional medicine to affect the functions of the intestinal barrier. However, based on our current understanding and the limited space of the paper, we only discuss several mechanisms that play the most important role in intestine protection. Similar to the discussion of the mechanisms, the signaling pathways or activating molecules stimulated by traditional medicine we discussed here are also a small part of the factors that may participate in the procedure of intestinal barrier protection by traditional medicine.

Although all of these studies suggest an effective role of traditional medicine in alleviating colitis symptoms, it should be noted that some traditional drugs may not generally surpass the suppressing effect of the active phase of UC compared to the contemporary developed molecular targeting drugs. The major advantage of traditional medicine may be reflected in its promising value to prolong the remission state and reduce relapse rate. An instance that supports this speculation was provided by the clinical trial of germinated barley foodstuff (GBF) in UC, showing that GBF could prolong remission of UC patients, a consequence that may be associated with the potential effect of GBF on maintenance or restoration of membrane barrier of the intestine (Hanai et al., 2004). Meanwhile, the application of some traditional medicine in UC is limited by the undetermined safety and bad bioavailability, a problem that may be largely solved by linkage with other water-soluble compounds or combination with nanocarriers or adjuvant compounds. Moreover, the efficacy of traditional medicine may be improved by the purification of effective compounds and the exclusion of toxic ingredients. More importantly, toxicity data need to be included in future *in vivo* studies, which may contribute to the estimation of MRSD for human clinical studies based on data obtained from animal studies.

As we have discussed in most cases, the barrier-protecting mechanisms may not be affected by the decoctions or compounds through direct interactions. The decoctions/compounds may first interact with various intracellular molecules and affect many signaling pathways, which regulate various biological processes and influence the intestinal barrier function. While it should be noted that the mechanisms employed by a particular decoction or a compound to exert its anti-UC effects are usually diverse. One decoction may contain thousands of compounds and target numerous molecules and pathways. Thus, it is not strange if other new targets or pathways were identified in future investigations. Moreover, as mentioned above, further identification of decoction ingredients and their immunological roles for intestinal barrier integrity is an important issue in the research of UC treatment. In addition, the findings of new targets and mechanisms for UC treatment in animal models may promote the development of new anti-UC medicine.
